# Obesity, smoking habits, and serum phosphate levels predicts mortality after life-style intervention

**DOI:** 10.1371/journal.pone.0227692

**Published:** 2020-01-16

**Authors:** Lena Håglin, Birgitta Törnkvist, Lennart Bäckman

**Affiliations:** Department of Public Health and Clinical Medicine, Family Medicine, Umeå University, Umeå, Sweden; Universidad Miguel Hernandez de Elche, SPAIN

## Abstract

**Background:**

Life-style interventions, including smoking cessation and weight control are of importance for managing future escalating prevalence of obesity. Smoking habits and obesity have jointly great impact on mortality, however mechanisms behind the effect and variables involved in the obesity paradox is still unknown.

**Objectives:**

This study examines risk factors for all-cause, cardiovascular, and cancer mortality in males and females with high cardiovascular risk, mediated by smoking habits, body mass index (BMI, kg/m^2^), and serum phosphate (S-P) levels.

**Methods:**

Patients were admitted to the Vindeln Patient Education Center in groups of 30 for a four-week residential comprehensive program (114 hours) focusing on smoking cessation, stress reduction, food preferences and selections, and physical exercise. The follow-up, in years from 1984 to 2014 corresponds to 30 years. This study included 2,504 patients (1,408 females and 1,096 males). Cox regression analysis was used to assess mortality risk associated with smoking habits, low and high BMI, and low and high S-P levels.

**Results:**

High BMI (>34,2 kg/m^2^), current smoking, type 2 diabetes mellitus (T2DM), high serum calcium (S-Ca), mmol/L and high systolic blood pressure (SBP, mmHg) were associated with all-cause mortality irrespective of sex. Former and current smoking females had a high all-cause mortality (adjusted hazard ratio [HR] 1.581; 95% CI 1.108–2.256, adjusted hazard ratio [HR] 1.935; 95% CI 1.461–2.562, respectively) while current smoking and high BMI increased risk for cardiovascular mortality (adjusted hazard ratio [HR] 3.505; 95% CI 2.140–5.740 and [HR] 1.536; 95% CI 1.058–2.231, respectively). Neither low nor high levels of S-P predicted all-cause, cardiovascular disease (CVD) and cancer mortality in males or females while low levels of S-P predicted all-cause mortality in smokers (adjusted hazard ratio [HR] 1.713; 95% CI 1.211–2.424). In non-smokers, low BMI (<27.6 kg/m^2^) was protecting and high BMI a risk for all-cause mortality. In males, ischemic heart disease (IHD), and low serum albumin (S-Alb) were associated with all-cause mortality. In females, an interaction between high BMI and smoking (HbmiSM) decreased the cardiovascular mortality (adjusted hazard ratio [HR] 0.410; 95% CI 0.179–0.937, respectively).

**Conclusions:**

High BMI and current smoking were associated with all-cause mortality in both males and females in the present high cardiovascular-risk cohort. In current smokers and non-smokers, T2DM and high S-Ca were associated with an increase in all-cause mortality, while low S-P was associated with all-cause mortality in smokers. Interaction between high BMI and smoking contribute to the obesity paradox by being protective for cardiovascular mortality in females.

## Introduction

The obesity related increase in mortality can be associated with low physical activity and/or smoking habits [1,2]. Smoking and obesity are independent predictors of mortality but the combination of current or recent smoking with a body mass index (BMI, kg/m^2^) ≥35 was shown to be related to an even stronger mortality risk [1]. Therefore, understanding how obesity and smoking habits interact can help to improve prediction of life expectancy [3, 4]. The association of BMI with mortality varies according to smoking status, age, and cause of death. Current and former smokers are at higher mortality risk than non-smokers [2]. Furthermore, normal weight smokers have higher risk of all-cause mortality than obese former smokers [5]. Interestingly, Badrick et al. (2017) concluded that smoking habits and associated metabolism contributed to the obesity paradox [6].

The prevalence of obesity is escalating in most populations in the world and several risk patterns, including smoking, contribute to mortality [7]. A synergistic effect from smoking and obesity on public health indicates a need for preventive strategies for maintaining good cardio-respiratory health [8]. A U- or J- shaped risk pattern of body weight and mortality indicate risk with both low and high BMI [9], which may be associated with previous diseases and smoking habits [10, 11]. With increasing age, being overweight may be positive for longevity [12]. Assessing weight loss and smoking habits in elderly with cardiovascular disease can increase knowledge about the obesity paradox. In addition, the study on sex differences and mortality necessitates an evaluation of U-shaped pattern of risk from BMI in addition to smoking habits. The influence from smoking on mortality can be associated with either high or low BMI and high or low serum phosphate (S-P) and by an association between mortality and phosphate.

High BMI is related to low S-P [13–15] whereas smoking might explain a high level of S-P [16–18]. Campos-Obando et al (2018), reported a higher S-P in smokers associated with mortality in males but not in females [15]. The direct effect of high levels of S-P on mortality has been reported in different diseases and clinical conditions [18–19] and even in adults with normal kidney function [20]. Low levels of S-P are also associated with increased mortality [21–22]. In addition, S-P levels decrease with increasing age in males, but not in females, which might express sex differences in relation to obesity and mortality.

Together this indicate the need of stratification for sex in studies of mortality associated with obesity and cardiovascular disease [23] and to include both low and high levels of S-P in studies of morbidity and mortality [24].

This study examines risk factors for all-cause, cardiovascular, and cancer mortality in males and females with high cardiovascular risk associated with smoking habits, BMI, and S-P levels. These individuals were admitted to a lifestyle intervention program. We hypothesized that mortality increases due to a disturbed phosphate metabolism expressed by a high S-P level in smokers.

## Material and methods

### Recruitment of patients

In this study, data collected in the cohort study undertaken as part of a lifestyle intervention program between 1984 and 1997 was analysed. The intervention and short-term follow-up have been previously described [25]. The program encouraged primary health care physicians in Västerbotten County (Sweden) to refer patients with a risk of contracting cardiovascular disease to the Vindeln Patient Education Centre. Eight new groups of patients were admitted each year. Body weight, blood pressure, and several other physical and biochemical variables were measured at the beginning of the residential period and at follow-up. A total of 107 groups of patients were referred to the Vindeln Centre, and the project was concluded in April 1997. The latest data point analysed in this study were collected from the follow-up of group 92 in December 1995.

### Studied population

This study included 2,504 patients (1,408 women and 1,096 men) who were at high risk of contracting cardiovascular disease. They were referred because traditional counselling and pharmacological treatment had failed to produce the expected result. We completed the original data files, as far as possible, with salient measurements from patient journals. Information concerning mortality was added to the data file from the Cause of Death Register, Statistics Sweden (28 August 2014).

The four main diagnoses represented in the cohort were hypertension (HT), type 2 diabetes mellitus (T2DM), ischaemic heart disease (IHD), and obesity/dyslipidaemia (Ob/Dys-lip). Table 1 lists the patients characteristics for each sex: age at first visit to the centre, blood variables, and number of patients deceased/alive in 28^th^ of August 2014, (n). Table 2 lists the diagnosis for patients at time of admittance and prevalence of deceased/alive (n, %) for each sex.

**Table 1:** Risk factors at baseline (mean±sd) for total, deceased and alive male and female patients at 28^th^ of August 2014.

	Total population		P-value	Deceased		P-value	Alive		P-value
	Males N = 1096	Females N = 1408		Males N = 552	Females N = 466		Males N = 544	Females N = 942	
Age, years	50.8±9.4	50.1±10.7	0.073	54.8±8.3	56.5±8.2	**0.002**	46.7±8.6	46.9±10.3	0.760
BMI, kg/m^2^	30.7±4.9	31.5±5.7	**0.000**	30.7±4.9	32.0±5.9	**0.000**	30.8±4.9	31.3±5.5	0.086
SBP, mmHg	148±19	145±19	**0.000**	152±19	151±19	0.785	145±18	143±19	**0.003**
S-TG, mmol/l	2.80±2.2	2.20±1.5	**0.000**	2.92±2.15	2.58±1.98	**0.008**	2.69±2.20	1.98±1.13	**0.000**
S-Chol, mmol/l	6.70±1.4	6.60±1.4	0.186	6.73±1.47	7.03±1.56	**0.002**	6.67±1.41	6.43±1.24	**0.001**
S-Alb, mmol/l	44.0±2.8	43.1±3.0	**0.000**	43.7±2.8	42.9±3.1	**0.000**	44.4±2.8	43.2±2.9	**0.000**
S-P, mmol/l	0.98±0.20	1.05±0.21	**0.000**	0.97±0.19	1.07±0.24	**0.000**	0.99±0.21	1.05±0.19	**0.000**
S-Ca, mmol/l	2.33±0.94	2.35±0.99	**0.000**	2.34±0.10	2.38±0.10	**0.000**	2.33±0.08	2.33±0.09	0.120

**Table 2:** Diagnosis for patients at time of admittance to the VPE-center and prevalence of deceased/alive with (%) presented for each sex.

Diagnosis	Males deceased/alive (% deceased)	Females deceased/alive (% deceased)
All patients in the study	552/544 (50.3)	466/942 (33.1)
Hypertension	261/313 (45.5)	251/532 (32.1)
Type 2 diabetes mellitus	168/81 (67.5)	136/111 (55.0)
Ischemic heart disease	67/36 (65.0)	22/25 (46.8)
Obesity/Dyslipidemia	36/106 (25.4)	38/241 (13.6)
Other diagnosis	17/8 (68.0)	19/33 (36.5)

The male/female ratio in all patients studied was 0.78 (1096/1408). The relative contribution of males and females (M/F) in the three smoking groups were, 167/136 = 1.23; 244/279 = 0.88; 685/993 = 0.69. More males than females in former smoker group while more females than males in current and non-smoker groups. The distribution of deceased /alive in % (n/n) in former, current and non-smokers was 31% (94/209), 40% (212/311) and 42% (712/966) respectively.

### Metabolites and physical examination

A full description of methods for laboratory analysis and for anthropometric and physical assessments are included in the study plan (http://dx.doi.org/10.17504/protocols.io.4wrgxd6).

In short, at start of the intervention programme, all patients provided fasting state (morning) blood samples. These samples were analysed according to standardized routine procedures of the Department of Clinical Chemistry, University Hospital, Umeå, Sweden. The levels of S-P, serum calcium (S-Ca), serum-cholesterol (S-Chol), and serum triglycerides (S-TG) were determined by routine methods using Hitachi 717 multianalyser (Boehringer Mannheim Diagnostica, Mannheim, Germany). Serum phosphate (S-P) was measured by a method based on the reduction of a phosphomolybdic acid complex with ammonium-iron (II)-sulphate, and total calcium was determined colorimetrically after the formation of a complex with orto-cresolphtalein. Systolic blood pressure (SBP) and body mass index (BMI) were registered. Serum albumin (S-Alb) was analysed using Vitros ALB slides on an Ortho Vitros 5.1 FS analyser.

### Statistical methods and models

Differences of mean for continuous data at baseline between males and females were tested with unpaired student’s t-test. The study population was stratified according to sex and smoking habits to explore whether the obesity–mortality relationship differed between former, current and non-smokers. Chi-square test was used to analyse distribution of males/females and deceased/alive in the three smoking groups. P-values <0.05 were considered statistically significant.

Cox regression was used to determine all-cause, cardiovascular and cancer mortality for males and females separately. Smoking habits were included as a risk and presented for former and current smokers with non-smoker as the referent population. The models are based on variables presented in Tables 1 and 2.

Survival analysis was performed, using time on study as the dependent variable, deceased or alive on 28 August 2014, to estimate the HR (95% CL) of all-cause, cardiovascular, and cancer mortality for different diagnoses and risk factors. No control group (i.e., a healthy group) was available.

Three levels of S-P (>1.11 mmol/l; 0.89–1.11 mmol/l; < 0.89 mmol/l) and three levels of BMI (kg/m^2^) (27.6–34.2; < 27.6; > 34.2) were used in the Cox regression. The survival time is presented in Kaplan Meier curves in Fig 3, Fig 4, and Fig 5.

Missing values were estimated as the mean of the corresponding variable. This gives an under-estimation of the variation in the variables, but a more accurate calculation of the HR than if missing values had not been estimated at all [26]. For each patient, a maximum of four missing values were permitted except for main diagnosis and smoking habits. Patients with missing values for main diagnosis or smoking habits or with more than four missing values in any of the other variables were excluded from the study. Percent of missing for each variable was less than 8,8 for males and 9,9 for females. The models estimated with and without missing gave no difference in the inference. The program Predictive Analytics Software (PASW Statistics, version 18.0.3 SPSS Inc., Chicago IL, USA) was used for the analysis.

### Ethical consideration

The Ethics Committee of Northern Sweden at the University of Umeå (Umeå, Sweden) approved the protocol on 22 November 2006 (Dnr 05–177 M). Trial registration: The study has been registered as a sub-study to the Lifestyle Intervention Trial no. ISRCTN79355192.

## Results

Differences in risk factors at baseline, between males and females for total, deceased and alive patients are presented in Table 1. A significantly lower S-P was shown in males as compared with females in the total patient population and when comparing deceased and alive subgroups (Table 1). We could observe, a higher age (p<0.000), SBP (p<0.000), and S-Ca (p = 0.003) in deceased males and females, compared with corresponding patients alive. Furthermore, a higher BMI (p = 0.024), S-TG (p<0.000), and S-Chol (p<0.000) were detected for deceased females compared with females alive and lower S-Alb (p<0.000) in deceased males as compared with males alive. A high prevalence (%) of deceased patients was found among the diagnoses of T2DM (M/F; 67.5/55.0) and IHD (M/F; 65.0/46.8) (Table 2).

High BMI (>34.2 kg/m^2^), current smoking, T2DM, S-Ca and SBP mmHg increased risk for all-cause mortality in both males and females (Table 3). Neither S-TG, low or high S-P, nor low BMI was associated with all-cause mortality risk. High S-Ca increased all-cause mortality, strongest in former and current smokers (Table 4). In females, both former and current smoking are risk factors for increased all-cause mortality and current smoking for cardiovascular mortality. High BMI indicate a high mortality risk while low BMI was protective for non-smokers. Low S-P and either T2DM or IHD diagnosis in current smokers and high BMI and T2DM in non-smokers predicted high risk for all-cause mortality. In non-smokers, an interaction between sex and T2DM show higher all-cause mortality from T2DM in females than in males (Table 3).

**Table 3:** Cox regression; HR (95%CL) for all-cause, CVD and cancer mortality (n = deceased).

	All-Cause		CVD		Cancer	
	MALES n = 552	FEMALES n = 466	MALES n = 283	FEMALES n = 203	MALES n = 110	FEMALES n = 124
Age, years	**0.840 0.756–0.932**	**0.792 0.716–0.875**	**0.695 0.585–0.825**	**0.561 0.443–0.710**	**0.670 0.489–0.918**	**0.605 0.419–0.873**
Age^2^	**1.001 1.000–1.002**	**1.002 1.001–1.003**	**1.002 1.001–1.004**	**1.004 1.002–1.006**	1.002 1.000–1.005	**1.003 1.000–1.007**
T2DM	**1.931 1.589–2.345**	**2.542 2.047–3.158**	1.108 0.823–1.492	**1.889 1.327–2.690**	1.485 0.888–2.483	1.166 0.696–1.953
IHD	**1.544 1.176–2.026**	1.341 0.860–2.092	1.153 0.784–1.697	1.654 0.907–3.017	0.854 0.389–1.873	0.642 0.205–2.006
Former smoking	0.790 0.597–1.044	**1.581 1.108–2.256**	1.315 0.876–1.975	1.907 0.965–3.769	**2.418 1.070–5.464**	1.106 0.596–2.052
Current smoking	**1.284 1.007–1.637**	**1.935 1.461–2.562**	1.274 0.899–1.805	**3.505 2.140–5.740**	1.339 0.768–2.334	1.832 0.988–3.395
Low BMI <27.6 kg/m^2^	0.850 0.693–1.043	0.992 0.776–1.268	0.923 0.672–1.267	1.065 0.700–1.619	0.832 0.508–1.363	1.214 0.746–1.976
High BMI >34.2 kg/m^2^	**1.629 1.278–2.075**	**1.451 1.146–1.838**	1.281 0.904–1.816	**1.536 1.058–2.231**	0.780 0.415–1.469	1.154 0.686–1.941
Low S-P <0.89 mmol/L	0.900 0.744–1.089	1.239 0.966–1.588	0.960 0.734–1.256	1.097 0.739–1.629	0.993 0.611–1.616	1.262 0.757–2.105
High S-P >1.11 mmol/L	0.908 0.714–1.154	1.148 0.928–1.419	0.928 0.649–1.329	1.201 0.856–1.685	1.226 0.680–2.209	0.774 0.489–1.224
S-Ca mmol/L	**1.104 1.022–1.192**	**1.103 1.005–1.210**	0.996 0.565–2.549	0.943 0.816–1.089	1.138 0.837–1.548	1.133 0.895–1.434
S-Alb mmol/L	**0.960 0.929–0.991**	0.972 0.943–1.002	**0.950 0.905–0.996**	0.957 0.906–1.010	1.039 0.952–1.135	1.027 0.951–1.108
SBP mmHg	**1.007 1.002–1.011**	**1.008 1.003–1.013**	1.004 0.998–1.010	1.000 0.991–1.009	1.005 0.993–1.018	1.004 0.993–1.016
S-Chol mmol/L	1.022 0.955–1.093	**1.096 1.020–1.177**	1.039 0.945–1.143	1.085 0.960–1.226	1.013 0.866–1.186	0.960 0.822–1.120
S-TG mmol/L	1.042 1.000–1.087	1.047 0.994–1.102	1.023 0.944–1.108	1.037 0.955–1.127	**1.163 1.027–1.318**	0.940 0.792–1.117
HbmiSM	0.614 0.359–1.051	0.988 0.600–1.627	1.200 0.565–2.549	**0.410 0.179–0.937**	0.994 0.280–3.528	0.552 0.177–1.723

**Table 4:** Cox regression (HR; 95%CL) for all-cause mortality in former, current and non-smokers.

Variables	Former smokers N = 303	Current smokers N = 523	Non-smokers N = 1678	
Males/Females n/n (%)	167/136 (55)	244/279 (47)	685/993 (41)	P<0.000
Deceased/Alive n/n (%)	94/209 (31)	212/311 (40)	712/966 (42)	P = 0.001
Age, yrs	0.992 0.722–1.362	**0.810 0.699–0.938**	**0.809 0.740–0.884**	
Age^2^	1.000 0.997–1.003	**1.002 1.000–1.003**	**1.002 1.001–1.002**	
Sex Male = 0, Female = 1	0.691 0.436–1.095	**0.640 0.471–0.869**	**0.396 0.327–0.479**	
T2DM	1.527 0.910–2.563	**1.680 1.194–2.364**	**2.084 1.658–2.619**	
IHD	0.901 0.375–2.166	**2.291 1.462–3.589**	1.314 0.987–1.749	
Low BMI <27.6 kg/m^2^	1.124 0.633–1.998	1.237 0.895–1.711	**0.807 0.665–0.980**	
High BMI >34.2 kg/m^2^	1.407 0.843–2.349	1.433 0.991–2.071	**1.510 1.261–1.809**	
Low S-P <0.89 mmol/L	0.677 0.395–1.161	**1.713 1.211–2.424**	0.902 0.755–1.077	
High S-P >1.11 mmol/L	1.177 0.689–2.009	0.972 0.694–1.362	1.001 0.827–1.213	
S-Ca, mmol/L	**1.303 1.000–1.699**	**1.359 1.145–1.612**	**1.076 1.007–1.149**	
S-Alb, mmol/L	0.974 0.887–1.069	**0.951 0.905–0.999**	0.975 0.950–1.002	
SBP, mmHg	1.007 0.995–1.018	1.004 0.996–1.012	**1.008 1.003–1.012**	
S-Chol, mmol/L	1.013 0.846–1.211	**1.185 1.064–1.319**	1.027 0.969–1.089	
S-TG, mmol/L	**1.193 1.097–1.297**	**0.934 0.875–0.996**	**1.084 1.041–1.130**	
Sex * T2DM	**NA**	**NA**	**1.419 1.027–1.960**	

NA = not included in the final analysis

High BMI, T2DM and current smoking increased cardiovascular mortality in females (Table 3). In addition, in males but not in females high S-TG increased risk for cancer mortality. An interaction between high BMI and smoking (HbmiSM) was protective for cardiovascular mortality in females.

**Fig 1:**
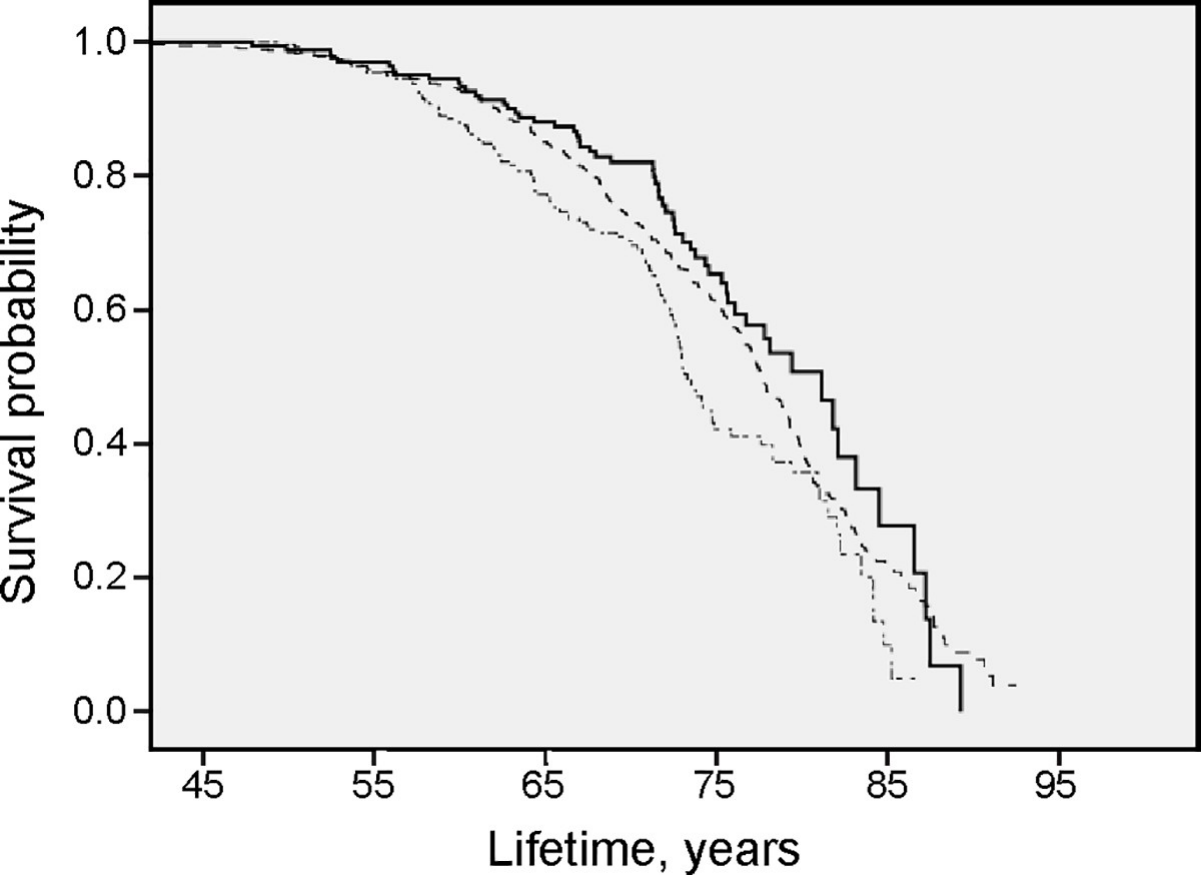
High all-cause mortality in male smokers. Survival probability for all-cause mortality in male *smokers* (∙─∙ ∙─∙ ∙─∙), *former smokers* (**──────**) and *nonsmokers* (-—-—-—-) illustrated with results from Kaplan-Meier analysis.

**Fig 2:**
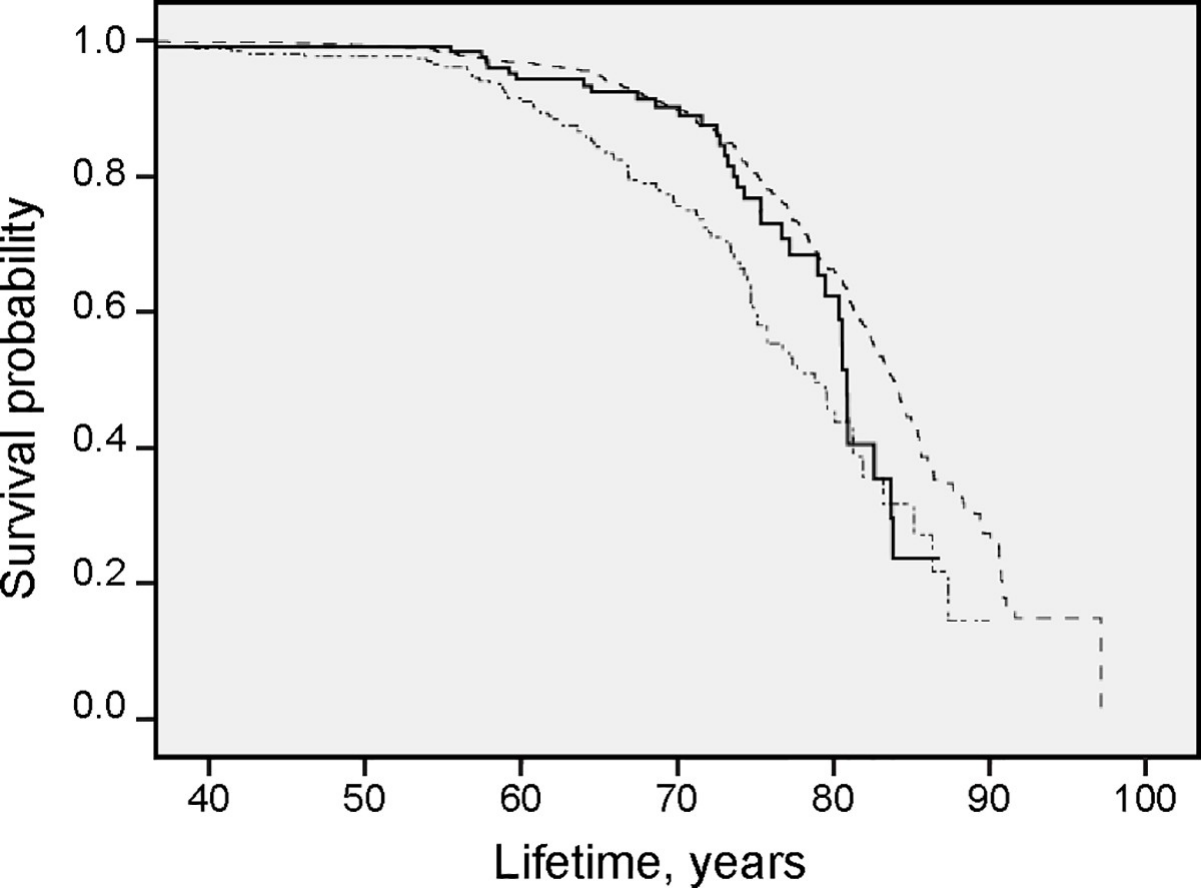
High all-cause mortality in female smokers. Survival probability for all-cause mortality in female *smokers* (∙─∙ ∙─∙ ∙─∙), *former smokers* (**──────**) and *nonsmokers* (-—-—-—-) illustrated with results from Kaplan-Meier analysis.

**Fig 3:**
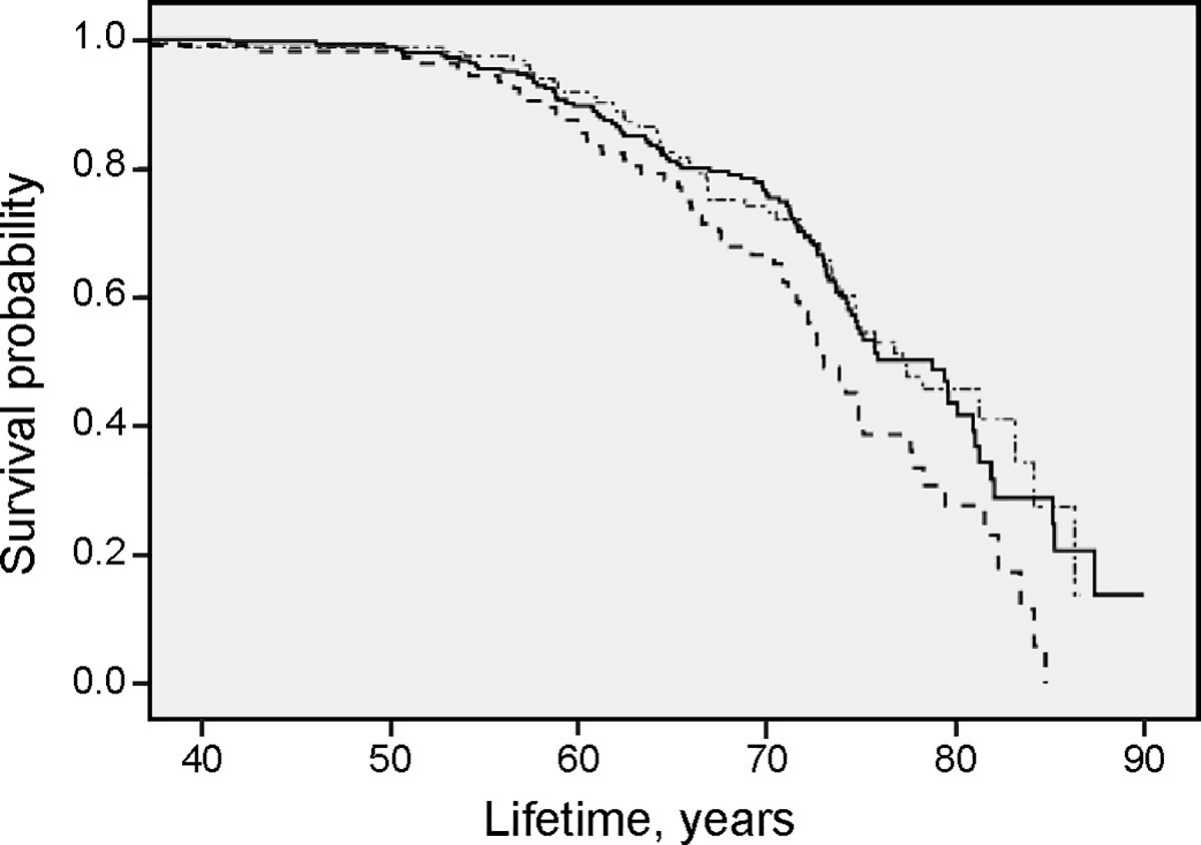
High all-cause mortality with a low serum phosphate level in smokers. Survival probability for all-cause mortality in total patient population. Smokers with *S-Phosphate* >1.11 mmol/l (∙─∙ ∙─∙ ∙─∙), *S-Phosphate* 0.89–1.11 mmol/l (**─────**) and *S-Phosphate* < 0.89 mmol/l (-—-—-—-) illustrated with results from Kaplan-Meier analysis.

**Fig 4:**
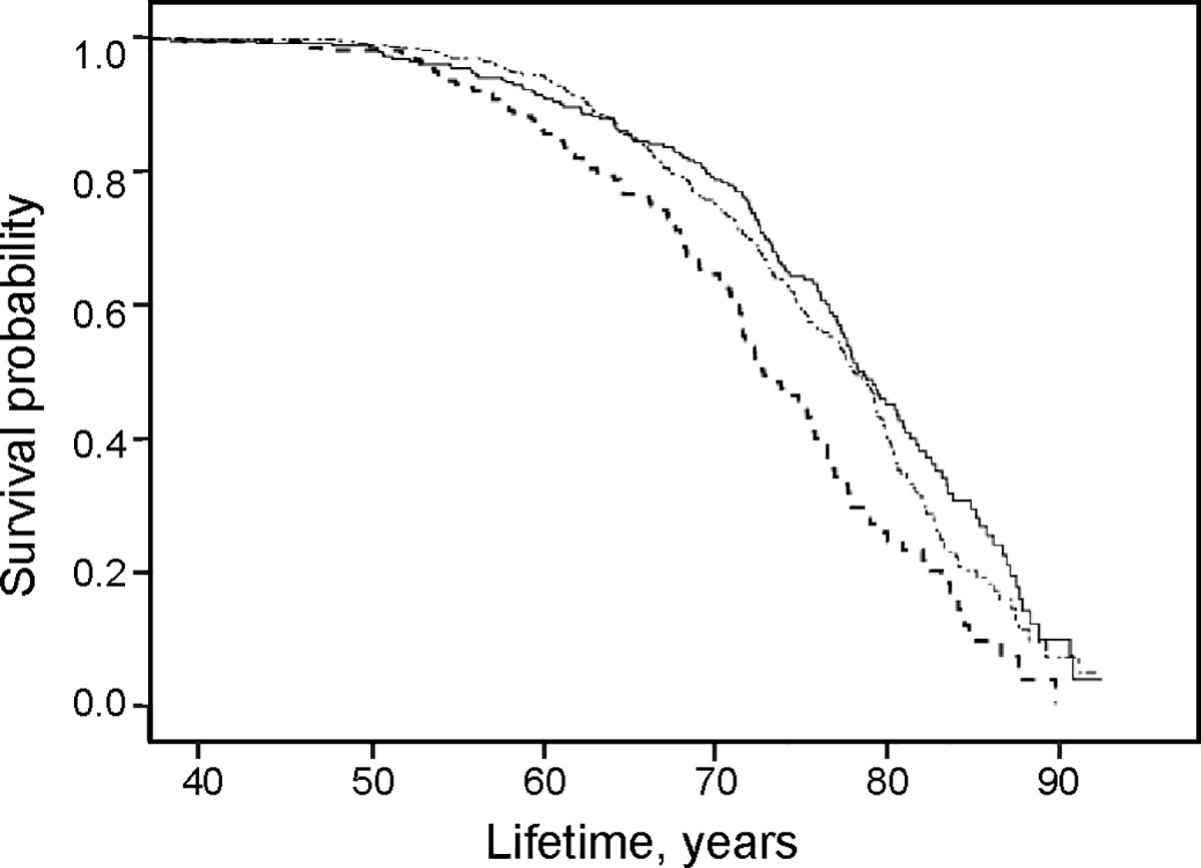
High all-cause mortality with a high BMI in males. Survival probability for all-cause mortality in males with *BMI 27*.*6–34*.*2* (∙─∙ ∙─∙ ─∙), BMI *< 27*.*6* (**─────**) and BMI *> 34*.*2* (-—-—- -) illustrated with results from Kaplan-Meier analysis.

**Fig 5:**
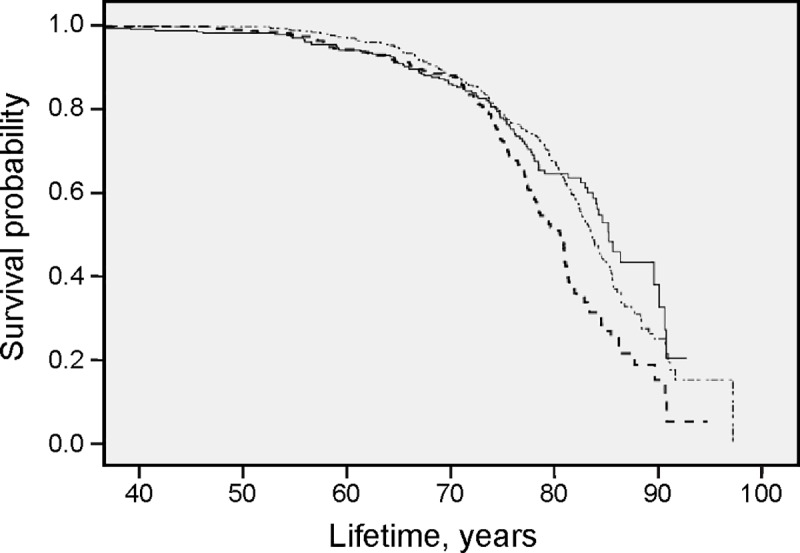
High all-cause mortality with a high BMI in females. Survival probability for all-cause mortality in females with *BMI 27*.*6–34*.*2* (∙─∙ ∙─∙ ∙─∙), BMI *< 27*.*6* (**─────**) and BMI *> 34*.*2* (-—-—- -) illustrated with results from Kaplan-Meier analysis.

Results from survival analysis in the total patient cohort for all-cause mortality are presented in the Kaplan Meier curves (Figs 1, 2, 3, 4 and 5). Smoking was associated with survival in both males (Fig 1) and females (Fig 2). In smokers, a low level of phosphate (< 0.89 mmol/L) was associated with reduced survival (Fig 3), and high BMI (>34.2) was associated with reduced survival in both males (Fig 4) and females (Fig 5).

## Discussion

The first finding from this study was that low S-P predicted mortality in smokers and thus our hypothesis that high phosphate levels increased mortality, must be rejected. The underlying mechanism of low S-P in smokers can be explained by smoking related habits, besides from obesity. We have previously shown an association between risk factors for cardiovascular disease including obesity and low phosphate levels in the present high-risk cohort [14]. Whether low S-P in smokers indicate a long-term effect from an imbalance in phosphate metabolism must be investigated further with information on renal losses and intracellular stores of phosphate.

Secondly, we have shown that high BMI was a stronger risk factor for all-cause mortality in non-smokers than in both former and current smokers and evident for both males and females. It has been concluded in another study that the mortality was lower in smokers with obesity than in non-smokers with obesity or normal weight [27].

Former and current smoking was independently associated with a higher all-cause and cardiovascular mortality in females but not in males. This might resemble a specific risk pattern in females associated with sarcopenic obesity, a condition where the subjects are obese with relatively low muscle weight. Information from BMI is not enough to conclude on body composition which requires measurements with body impedance or dual energy X-rays assessments. Further studies are thus needed to assess this higher mortality risk, comparing smoking in females with males and its association with the obesity paradox.

The combination of high BMI and smoking status contributes to high mortality risk through a synergistic effect [3, 28]. As we do not know how physical inactivity or obesity adds to the risk of smoking, stratification for these three adverse outcomes on health must be the most appropriate way, instead of adjusting for smoking, when investigating morbidity and mortality risk [29]. Adding risk factors may generate new errors if an interaction, for example, between obesity and smoking are stronger and more negative than obesity and physical activity for cardiovascular mortality. The confounding effects of smoking, IHD, and T2DM were adjusted for in the Cox regression of the present study.

In this study, an interaction variable, between severe obesity (BMI >34.2) and current smoking (HbmiSM), indicates protection from cardiovascular disease mortality in females, maintaining the high risk with both smoking and high BMI. The level of S-P in smokers may be the biomarker that explain the protection or risk of mortality in the obese patients. Narumi et al concluded that the advantages of obesity are not found in patients with the metabolic syndrome [30], which might support the hypothesis of high S-P as the active biomarker in the obesity paradox, since patients with risk factors in the metabolic syndrome suffer from low S-P [14]. We used BMI and levels of S-P in the analysis of mortality risk after stratification for sex. Using S-P levels, a marker for morbidity and mortality, necessitates stratification for sex as earlier studies report lower S-P levels in males than in females. Smokers have higher levels of S-P than non-smokers and former smokers [16], and for obesity there is a low level of S-P [13–15].

A typical cardiovascular risk pattern as predictors for mortality was shown in the present cohort of patients admitted for lifestyle intervention. The diagnosis before lifestyle changes indicate that obesity-related and diabetes-associated disturbances dominated. The patients were referred by the primary health care and regional hospital for the comprehensive lifestyle intervention. In previous studies, we reported that these patients with T2DM, high calcium, and low magnesium had increased mortality compared with patients with normal electrolytic levels, however, we did not adjust for smoking habits [31].

In the present study, former and current smokers had a high S-Ca, that predicted a high all-cause mortality. Both high S-Ca and low S-P were related to increased mortality and may represent a disturbance associated with smoking. The high risk for mortality with high S-Ca in current smokers, may be additive or multiplicative to the low S-P levels and explained by a disturbed regulation of S-Ca and S-P levels. By increase in S-P, the high risk from S-Ca may be reduced, which needs to be assessed in future studies. The prevalence of smokers increased six-fold from lowest (6.9%) to highest level of phosphate (40.1%) indicating an association between smoking and S-P levels but this was not considered in the discussion as a risk condition for cardiovascular events in that study [32].

In a randomized study on this cohort, an intervention effect over 18 years indicates benefits on lipid status, primarily from lowering of S-TG, which reduced the all-cause mortality [25]. Besides high S-Ca, S-TG predicted all-cause mortality in former smokers, in the present study. Smoking cessation and stress handling were part of the intervention that focused on physical activity and improved diet by selecting nutritious food groups, which together has a TG-lowering effect.

Low levels of S-P have been reported to increase mortality, a marker for malnutrition in an elderly population [21]. The reduction of S-P can be a consequence from refeeding or trauma, or due to disturbed energy balance in obesity. Other studies have found an association between hypophosphatemia and increased mortality [22], longer hospital stay [33], myocardial infarction, and mortality [34]. One possibility could be that a low S-P level in smokers may be linked to obesity, malnutrition, or diseases in addition to abuse of alcohol. When smoking increases S-P, large amounts of alcohol can result in losses of phosphate. In addition, the low S-P associated with obesity, might counterbalanced the high levels of S-P from smoking in obese patients.

To our knowledge, this is the first study to report on mortality from low and high phosphate in an obese cohort of patients with high cardiovascular risk and with smoking in focus. Thus, considering risk with high BMI and smoking, a conclusion about causes or consequences may be even more difficult to settle due to the associations with increased age due to mortality differences between males and females. Future studies regarding the dynamics of S-P levels can increase the understanding of the causes behind mortality due to smoking and/or high BMI. Adding information on the level of phosphate to the cluster of risk factors in obesity will increase knowledge about the additive effect from both smoking and T2DM on mortality. Moreover, elucidating the mechanism and impact of high S-P from smoking on mortality will generate information applicable for the obesity paradox. In smokers, a blocked energy generation in mitochondria due to low dissociation of oxygen result in hypoxia with reduced generation of ATP might be the underlying cause. Surprisingly, low level of S-P increased all-cause mortality in smokers in the present study, which might be explained by other factors contributing to the limitation of energy generation. A low level of phosphate due to transcellular shift as opposed to low levels due to losses by urine or low intake in addition to intracellular phosphate depletion most possible, makes the differences in mortality risk levels.

Several metabolic disturbances account for the relation between cardiovascular death and BMI, and obesity per se might not give rise to increased mortality. The high-risk profile in metabolic syndrome differs between males and females and the mortality rate is higher in males [35]. If disturbed phosphate metabolism is involved, the question to be raised is this: Which disturbances are most hazardous, low or high S-P levels? Since males have a different risk factor profile than females, as well as difference in longevity, it is important to study mortality for each sex separately and consider alcohol and smoking habits independently or as an interaction. The influence from sex differences in S-P, needs further exploration when studying obesity-related disturbances. Here, the aim was to investigate whether the level of phosphate, which is high in smokers, could predict mortality in the present cohort. It was, however, revealed that low S-P in smokers was associated with higher all-cause mortality. Taken together, consideration of either low or high S-P as a risk marker for mortality in obesity can contribute to the understanding of underlying mechanisms behind the obesity paradox.

## Supporting information

S1 File LML plots with legends. (DOCX)
