# Supplementary material for: Obesity, smoking habits, and serum phosphate levels predicts mortality after life-style intervention
Source: PLoS One. 2020 Jan 16;15(1):e0227692. doi: 10.1371/journal.pone.0227692 (PMC6964906; doi:10.1371/journal.pone.0227692)
Supplement: S1 File — (DOCX) [file pone.0227692.s001.docx]

Legends to LML plots

These graphs test the assumption of proportional Hazards by presenting log minus log of survival time (log Hazard) against lifetime. If the lines are parallel the assumption is fulfilled. If the hazard ratio (HR) is not significantly deviant from 1 the lines are attaching and sometimes crossing.

**Plot 1-3 for males (A) and females (B)** shows the mortality risk in former smokers compared to non-smokers and current smokers compared to non-smokers.

**All-cause mortality**

The HR for males is not significant for former smokers but significant for current smokers, HR=0,790 and 1,284, respectively. For females, HR is significant for both former and current smokers, HR=1,581 and 1.935, respectively.

**Plot 1A**

**Plot 1B**

**CVD mortality**

The HR for males is neither significant for former smokers nor for current smokers (HR=1,315 and 1,274, respectively). For females, HR is significant for current smokers but not for former smokers (HR=3.505 and 1.907, respectively).

**Plot 2A**

**Plot 2B**

**Cancer mortality**

The HR for males is significant for former smokers but not for current smokers (HR=2,418 and 1,339, respectively). For females, neither formers nor current smokers had significantly higher risk (HR=1.106 and 1.832, respectively).

**Plot 3A**

**Plot 3B**

**All-cause mortality**

**Plot 4 – 6** shows the mortality risk in females (= 1) against males (= 0) against in plot 4 for former smokers (HR = 0.691, non-sign), plot 5 for current smokers (HR = 0.640, sign) and plot 6 for non-smokers (HR = 0.396, sign).

**Plot 4**

**Plot 5**

**Plot 6**
